# Supplementary material for: Unveiling the Bioactive Potential of the Invasive Jellyfish Phyllorhiza punctata Through Integrative Transcriptomic and Proteomic Analyses
Source: Biomolecules. 2025 Aug 4;15(8):1121. doi: 10.3390/biom15081121 (PMC12383608; doi:10.3390/biom15081121)
Supplement: Supplementary file 1 [file biomolecules-15-01121-s001.zip › Suplementary Material/FileS3/PEPFOLD4/XP_065064308_8158/clusters.html]

 PEP-FOLD clusters


Download archive of all models  here   

|  |  |  |
| --- | --- | --- |
| # | file | sOPEP |
| # cluster 1 - Size: 11 |  |  |
|  | XP\_065064308\_8158-model1 | -28.9819 |
|  | XP\_065064308\_8158-model1.1 | -28.8072 |
|  | XP\_065064308\_8158-model1.2 | -28.356 |
|  | XP\_065064308\_8158-model1.3 | -27.8606 |
|  | XP\_065064308\_8158-model1.4 | -26.7849 |
|  | XP\_065064308\_8158-model1.5 | -26.2863 |
|  | XP\_065064308\_8158-model1.6 | -26.1301 |
|  | XP\_065064308\_8158-model1.7 | -25.8432 |
|  | XP\_065064308\_8158-model1.8 | -25.7295 |
|  | XP\_065064308\_8158-model1.9 | -25.2435 |
|  | XP\_065064308\_8158-model1.10 | -25.0743 |
| # cluster 2 - Size: 8 |  |  |
|  | XP\_065064308\_8158-model2 | -28.3407 |
|  | XP\_065064308\_8158-model2.1 | -27.3301 |
|  | XP\_065064308\_8158-model2.2 | -27.1097 |
|  | XP\_065064308\_8158-model2.3 | -27.0425 |
|  | XP\_065064308\_8158-model2.4 | -27.0125 |
|  | XP\_065064308\_8158-model2.5 | -26.7471 |
|  | XP\_065064308\_8158-model2.6 | -26.7293 |
|  | XP\_065064308\_8158-model2.7 | -25.6915 |
| # cluster 3 - Size: 1 |  |  |
|  | XP\_065064308\_8158-model3 | -27.7401 |
| # cluster 4 - Size: 5 |  |  |
|  | XP\_065064308\_8158-model4 | -26.8066 |
|  | XP\_065064308\_8158-model4.1 | -25.6779 |
|  | XP\_065064308\_8158-model4.2 | -25.6072 |
|  | XP\_065064308\_8158-model4.3 | -25.5891 |
|  | XP\_065064308\_8158-model4.4 | -25.2035 |
| # cluster 5 - Size: 27 |  |  |
|  | XP\_065064308\_8158-model5 | -26.1811 |
|  | XP\_065064308\_8158-model5.1 | -25.8581 |
|  | XP\_065064308\_8158-model5.2 | -25.7863 |
|  | XP\_065064308\_8158-model5.3 | -25.7621 |
|  | XP\_065064308\_8158-model5.4 | -25.6492 |
|  | XP\_065064308\_8158-model5.5 | -25.6462 |
|  | XP\_065064308\_8158-model5.6 | -25.5699 |
|  | XP\_065064308\_8158-model5.7 | -25.5052 |
|  | XP\_065064308\_8158-model5.8 | -25.5031 |
|  | XP\_065064308\_8158-model5.9 | -25.478 |
|  | XP\_065064308\_8158-model5.10 | -25.4211 |
|  | XP\_065064308\_8158-model5.11 | -25.3271 |
|  | XP\_065064308\_8158-model5.12 | -25.3243 |
|  | XP\_065064308\_8158-model5.13 | -25.3106 |
|  | XP\_065064308\_8158-model5.14 | -25.2782 |
|  | XP\_065064308\_8158-model5.15 | -25.2441 |
|  | XP\_065064308\_8158-model5.16 | -25.2375 |
|  | XP\_065064308\_8158-model5.17 | -25.194 |
|  | XP\_065064308\_8158-model5.18 | -25.0919 |
|  | XP\_065064308\_8158-model5.19 | -25.0894 |
|  | XP\_065064308\_8158-model5.20 | -25.0189 |
|  | XP\_065064308\_8158-model5.21 | -24.9803 |
|  | XP\_065064308\_8158-model5.22 | -24.9545 |
|  | XP\_065064308\_8158-model5.23 | -24.8907 |
|  | XP\_065064308\_8158-model5.24 | -24.8309 |
|  | XP\_065064308\_8158-model5.25 | -24.7514 |
|  | XP\_065064308\_8158-model5.26 | -24.6994 |
| # cluster 6 - Size: 12 |  |  |
|  | XP\_065064308\_8158-model6 | -26.136 |
|  | XP\_065064308\_8158-model6.1 | -26.0212 |
|  | XP\_065064308\_8158-model6.2 | -25.8947 |
|  | XP\_065064308\_8158-model6.3 | -25.6676 |
|  | XP\_065064308\_8158-model6.4 | -25.3686 |
|  | XP\_065064308\_8158-model6.5 | -25.315 |
|  | XP\_065064308\_8158-model6.6 | -25.2907 |
|  | XP\_065064308\_8158-model6.7 | -25.1575 |
|  | XP\_065064308\_8158-model6.8 | -24.9387 |
|  | XP\_065064308\_8158-model6.9 | -24.9242 |
|  | XP\_065064308\_8158-model6.10 | -24.8384 |
|  | XP\_065064308\_8158-model6.11 | -24.6321 |
| # cluster 7 - Size: 2 |  |  |
|  | XP\_065064308\_8158-model7 | -25.5717 |
|  | XP\_065064308\_8158-model7.1 | -25.5397 |
| # cluster 8 - Size: 8 |  |  |
|  | XP\_065064308\_8158-model8 | -25.5696 |
|  | XP\_065064308\_8158-model8.1 | -25.2252 |
|  | XP\_065064308\_8158-model8.2 | -25.1949 |
|  | XP\_065064308\_8158-model8.3 | -25.1216 |
|  | XP\_065064308\_8158-model8.4 | -24.9236 |
|  | XP\_065064308\_8158-model8.5 | -24.6377 |
|  | XP\_065064308\_8158-model8.6 | -24.6325 |
|  | XP\_065064308\_8158-model8.7 | -24.4467 |
| # cluster 9 - Size: 12 |  |  |
|  | XP\_065064308\_8158-model9 | -25.2955 |
|  | XP\_065064308\_8158-model9.1 | -25.1409 |
|  | XP\_065064308\_8158-model9.2 | -25.0962 |
|  | XP\_065064308\_8158-model9.3 | -24.9475 |
|  | XP\_065064308\_8158-model9.4 | -24.7706 |
|  | XP\_065064308\_8158-model9.5 | -24.6287 |
|  | XP\_065064308\_8158-model9.6 | -24.58 |
|  | XP\_065064308\_8158-model9.7 | -24.4993 |
|  | XP\_065064308\_8158-model9.8 | -24.4198 |
|  | XP\_065064308\_8158-model9.9 | -24.3991 |
|  | XP\_065064308\_8158-model9.10 | -24.2226 |
|  | XP\_065064308\_8158-model9.11 | -24.1677 |
| # cluster 10 - Size: 6 |  |  |
|  | XP\_065064308\_8158-model10 | -25.119 |
|  | XP\_065064308\_8158-model10.1 | -25.083 |
|  | XP\_065064308\_8158-model10.2 | -24.8534 |
|  | XP\_065064308\_8158-model10.3 | -24.7917 |
|  | XP\_065064308\_8158-model10.4 | -24.7592 |
|  | XP\_065064308\_8158-model10.5 | -24.7079 |
| # cluster 11 - Size: 1 |  |  |
|  | XP\_065064308\_8158-model11 | -24.8252 |
| # cluster 12 - Size: 1 |  |  |
|  | XP\_065064308\_8158-model12 | -24.6288 |
| # cluster 13 - Size: 2 |  |  |
|  | XP\_065064308\_8158-model13 | -23.9119 |
|  | XP\_065064308\_8158-model13.1 | -19.7774 |
| # cluster 14 - Size: 1 |  |  |
|  | XP\_065064308\_8158-model14 | -20.3484 |
| # cluster 15 - Size: 2 |  |  |
|  | XP\_065064308\_8158-model15 | -19.9709 |
|  | XP\_065064308\_8158-model15.1 | -18.2464 |
| # cluster 16 - Size: 1 |  |  |
|  | XP\_065064308\_8158-model16 | -18.1773 |
|  |  |  |
